# Exploring Women’s Decision-Making Power and HIV/AIDS Prevention Practices in South Africa

**DOI:** 10.3390/ijerph192416626

**Published:** 2022-12-10

**Authors:** Russell Kabir, Angi Alradie-Mohamed, Nahida Ferdous, Divya Vinnakota, S. M. Yasir Arafat, Ilias Mahmud

**Affiliations:** 1 School of Allied Health, Anglia Ruskin University, Chelmsford CM11SQ, UK; 2 Barts Health NHS Trust, London EC1A 7BE, UK; 3 Department of Nursing and Public Health, The University of Sunderland, London E14 9SG, UK; 4 Enam Medical College and Hospital, Dhaka 1340, Bangladesh; 5 Department of Public Health, College of Public Health and Health Informatics, Qassim University, Al Bukairiyah 52741, Saudi Arabia

**Keywords:** decision making, HIV, women, South Africa, prevention

## Abstract

Decisions regarding sexual and reproductive health significantly impact women’s health and their protection against HIV/AIDS and other sexually transmitted infections. These decisions also impact females’ ability to reach their reproductive goals. Women’s autonomy is recognized to be vital to women’s access to reproductive healthcare, the use of contraceptives, the capacity to avoid or receive treatment for STIs (including HIV), and other reproductive and sexual health issues. This research investigated the association between the decision-making power of South African women (of reproductive age) and their knowledge and practices regarding HIV/AIDS preventive measures. The present study used data from the South Africa Demographic and Health Survey 2016. A total of 8514 women aged 15–49 years who participated in the survey were used for this research. The mean age of the women was 30.21 years, with an SD of 9.86. Approximately 38.5% of the women decided on contraceptive use, and only 11.7% of women’s partners and 49.8% of respondents were jointly involved in the decision-making process of contraceptive use. All HIV preventive measures under study were statistically significantly associated with high decision-making power; the use of a condom by the husband or partner of the women was the most significant; husbands or partners of the women with high autonomy were three times more likely to use condoms.

## 1. Introduction

Decisions regarding sexual and reproductive health significantly impact women’s health and protection against human immunodeficiency viruses, acquired immune deficiency syndrome (HIV/AIDS), and other sexually transmitted infections (STIs). These decisions also impact women’s ability to reach their reproductive goals. It should be noted that women are disproportionately infected and affected by HIV/AIDS epidemics [1]. Since 1994 after the adaptation of the International Conference on Population and Development (ICPD) program of action, women’s empowerment and autonomy have been recognized to be vital to women’s access to reproductive healthcare, the use of contraceptives, capacity to avoid or receive treatment for STIs (including HIV) and other reproductive and sexual health issues [2,3]. Despite women empowerment movements, women’s participation in reproductive health decisions varies due to social and cultural factors related to patriarchy and gender inequality [4,5,6]. Gender power inequities significantly affect women’s power in sexual relationships and are thought to have a prominent role in increasing women’s risk of HIV infection [7,8].

Individual factors also contribute to women’s decision to use contraceptives, such as age, material status, employment status, income, ethnicity, history STIs or unwanted pregnancies [9], occupation, religion, duration of the relationship, home language, perception on male spouse right to sex, forced sex and sexual inter-relationship communication [10]. For most married women, their husbands are the primary source of STIs. Thus the inability of some wives to negotiate safer sex with their husband with multiple partners significantly impact the transmission of HIV/AIDS [11]. A study in Kenya [12] reported that among couples with an HIV-seropositive husband and HIV-seronegative wife, the decision for either partner to start antiretroviral therapy (ART) or prep treatment is entirely controlled by the husbands. Authors emphasize that women struggle to negotiate safer sex when adopting HIV-prevention strategies against men’s sexual desires [12].

Dayson and Moore (1983) defined autonomy as the ability to obtain and use information to make decisions regarding individuals’ private matters [13]. Women who have high autonomy regarding household decisions tend to be able to take their own healthcare decisions [14]. However, reproductive healthcare decisions differ from other female healthcare decisions as they concern the male partner/spouse (engaging in sexual intercourse and using contraceptives or condoms). Women’s ability to make reproductive and sexual health decisions can reduce their vulnerability to STIs, including HIV/AIDS, from infected sexual partners [15]. Women’s autonomy is significant in sexual health; women with high decision-making power can negotiate safer sex in risky situations and thus protect themselves from HIV/AIDS [16].

Sumri (2015) found a positive association between women’s decision-making autonomy and the use of contraceptives [17]. Similar results were reported by other studies [18], which additionally found a positive association between women’s decision-making autonomy and the ability to negotiate sexual activity and agreement on fertility preferences. Different studies reported the association between women’s autonomy and HIV/AIDS knowledge and preventive practices [16,19]. At the same time, a cohort study among young South African women found that relationship inequity increases the risk of HIV infection [20].

In South Africa, HIV/AIDS represents a significant public health concern. In 2016, 19% of South African adults (15 years and older) were living with HIV, with HIV prevalence higher among females (23%) than males (13%), according to the South Africa Demographic and Health Survey 2016 [21]. In 2018, the number of South Africans living with HIV reached 7,700,000 individuals, with women being more affected by the disease. The prevalence of HIV among females 15–49 years was 25.8% in comparison to 15% among males of the same age group, while new HIV infection was more than double among young females (15–24 years) in comparison to males in the same age group that year [22].

South Africa has made major efforts through the years for the treatment and prevention of HIV; programs for the prevention of HIV, including Prevention of mother-to-child transmission (PMTCT), condom distribution, Pre-exposure Prophylaxis (PrEP) program, and HIV awareness and education programs [23]. This study uses published data from the South Africa Demographic and Health Survey 2016 [21] to investigate the association between the decision-making power of South African women (of reproductive age) and their knowledge and practices regarding HIV/AIDS preventive measures.

## 2. Materials and Methods

### 2.1. Design

The present study used data from the South Africa Demographic and Health Survey 2016. The survey used a 2-stage stratified cluster sampling approach. Following the census 2011, at the first stage, primary sampling units (PSUs) were formed with a probability proportional to size formula, where enumeration areas (EAs) were treated as primary sampling units. In the second stage, dwelling units (DUs) were selected using systematic sampling. A total of 750 PSUs were selected from 26 strata, and a fixed number of 20 DUs from each cluster were selected in an organized fashion. Finally, 15,292 households were selected for the survey, and 11,083 households participated, yielding a response rate of 83%. A total of 8514 women aged 15–49 years who participated in the survey were included in this research. All the women included in this study were married at the time of the survey or were living together with a partner.

### 2.2. Measures

#### 2.2.1. Statistical Methods

For constructing the variable decision-making power, the following questions were asked to women: (i) the decision-maker for using contraception, (ii) the person who usually decides on respondent’s healthcare, (iii) the person who usually decides on large household purchases (iv) person who usually decides on visits to family or relatives (v) person who usually decides how to spend respondent’s earnings. The answers were coded from 1 to 3, such as respondents were coded as 1, the husband, someone else and others were coded as 2, and the respondent and husband/partner were coded as 3. For this study, a score of 5 (the highest decision-making score) was allocated to the answering respondent when the respondent herself was responsible for taking the decision, a score of 4 was assigned when the respondent and husband/partner jointly took the decision. The scores of 3,2 and 1 (lowest decision-making score) were allocated when the decision was taken by the husband or partners, someone else and others, respectively.

The scores of the above questions were added together to construct the variable decision-making power, which varied from 5 to 24. The total scores were divided into three categories: no decision-making power was considered for scores between 5 to 10, moderate decision-making power was defined as scores between 11 to 15 and scores 16 or above was regarded as high decision-making power.

The dependent variable of this research is knowledge and practices regarding HIV/AIDS preventive measures. This variable was created by combining–(i) condom used during last sex, (ii) having ever been tested for HIV, (iii) drugs used to avoid HIV transmission during pregnancy, and (iv) knowledge and use of HIV test kits. These variables were determined by respondents answering “No” to any of the string questions, and it was coded as “0,” and respondents answering “Yes” were coded as “1.”

The controlled variables used in this research are-

I. age group: participants’ current age was categorized into four main categories such as 15–24 years, 25–34 years, 35–44 years, and 45 years and above;
II. The place of residence is divided into urban and rural;
III. Educational status is categorized into–no education, primary education, secondary education, and higher education;
IV. Ethnicity was divided into black/African, white, colored, Indian/Asian and other
V. Employment status with “yes” and “no” categories;
VI. Reading the newspaper, listening to the radio and watching television were categorized as “not at all,” “less than once a week,” and “at least once a week”;
VII. The wealth index variable is measured based on possession of household assets, and scores were given using principal component analysis, and the categories were: poorest, poorer, middle, richer, and richest.

#### 2.2.2. Analysis

Both descriptive and bivariate logistic regression analyses were performed using IBM SPSS (Statistical Package for the Social Sciences) version 26. The association between the dependent and independent variables was measured using odds ratios with a 95% confidence interval. Statistical significance was considered at a 5% level.

### 2.3. Ethical Aspects

As the data for this research was obtained from a publicly available source, no institutional review board approval was sought.

## 3. Results

Summary information for the selected controlled variables used in the odds ratio analysis is presented in Table 1. The mean age of the women is 30.21 years, with an SD of 9.86. About 56.4% of participants are from urban areas, 77.3% of women completed secondary education, and the majority (86.4%) are Black or African women. Only 32.2% of women were employed during the survey, and 23% were from middle-income households. Logistic regression analyses were performed among the explanatory variables and condom use, tested for HIV, drugs used to avoid HIV transmission and use of HIV test kits. According to Table 1, the age group, place of residence and ethnicity variables were negatively and educational status, employment status, and reading newspapers or magazines were positively associated with condom use as a prevention method for HIV. The dependent variable tested for HIV was significantly associated with the older age group, higher education, being employed, poorest rather than richest in wealth index and Black African ethnic group compared to White and Asian ethnic groups. Additionally, women from rural area have a 16% lesser chance of going for HIV testing. Prevention of HIV transmission during pregnancy through drug use is significantly associated with age group, employment status, and reading the newspaper at least once a week. However, the drug use odds were lower among white, colored, and other ethnic groups compared to the black African ethnic reference groups.

The use of HIV test kits is negatively linked with place of residence; rural women are 22% less likely to use HIV test kits. However, a positive association between using HIV test kits is noticed with wealth index, employment status, and reading newspapers. The respondents with higher education have a two times higher chance of using the HIV test kit than those without no education. The odds of using an HIV test kit are higher among individuals who listen to the radio at least once a week. Television watching less than once a week is associated with using an HIV test kit. Women with higher education have two times higher odds of using HIV test kits than women without education. Also, women of 45 years and over have lower odds of using HIV test kits than women of 15 to 24 years. Among all the ethnic groups, colored ethnic group women have 22% lower probability of using the HIV test kit.

The autonomy scores were compared with HIV prevention strategies adopted by the participants (Table 2). The decision-making score was categorized -as low, moderate, and high. Using condoms as an HIV prevention strategy shows a significant positive association with a high decision-making score. Women with high decision-making power have a three times higher chance of using condoms as a prevention method for HIV than women with low decision-making power. Women with high decision-making power also show a negative association with using drugs for HIV transmission prevention during pregnancy, testing for HIV, and using HIV test kits.

HIV prevention and individual decision-making score comparison using odds ratio analysis are shown in Table 3. Approximately 38.5% of women have the freedom to decide on contraceptive use, and only 11.7% of women’s husbands or partners, someone else and others is involved in the decision-making process of contraceptive use. It has been found that women who decide on healthcare issues with their husband or partner and others are five times more likely to use drugs for HIV prevention than women who choose their health care matters. About 20.3% of women can decide on large household purchases without involvement from husbands, partners, or others. The husband or partner’s decision on the respondent’s visit to family or relatives is negatively associated with using drugs to avoid HIV transmission. The odds are two times higher for HIV testing among the respondent when the respondent and their husband or partner are involved in the decision-making process of the respondent’s earnings, compared to the women who take their own decision with their earnings.

## 4. Discussion

This study examined the association between women’s autonomy and their practices regarding HIV prevention; the use of condoms, getting tested for HIV, drugs used to avoid HIV transmission during pregnancy, and knowledge and use of HIV test kits. High decision-making power among women was statistically significantly associated with the help of all preventive measures for HIV. Women with high decision-making power were three times more likely to use condoms as preventive methods for HIV prevention when compared to women with low decision-making power. A different finding was reported in a study in India, where women with less autonomy were more likely to be aware of using condoms [19]. Some women in relationships exhibit sole control over contraceptive use. Still, some women believe in a shared decision-making process with their husbands or partner or allow their husbands or partners to choose for them [9]. It is established that condom use reduces the transmission of HIV and other STIs when used correctly and regularly [24].

The study sample had high decision-making power when it came to using contraception, with 38.5% making decisions alone and 49.7% making the decision jointly with their husbands/partners or others. This is high in comparison to a study conducted in South Asia [25], where the percentage of women taking the contraceptive decision alone ranged from 28.1% in India, 17.6% in Bangladesh, 13.4% in Nepal, and 11.5% in Sri Lanka.

South African women are highly motivated to use modern contraceptive methods, and they take contraceptive decisions predominantly by themselves, except for using condoms. Still, many do not inform or consult their partner about those decisions [26]. However, they face difficulties negotiating using condoms with their partners [26], which supports the finding of this study that women need to have high autonomy to be able to insist on condom use. Acting on the decision to use condoms requires a degree of insistence, assertiveness, negotiation skills, and assessment of the outcome of those negotiations [27].

This study also supports that age is associated with the use of HIV preventive measures -except the use of the HIV test kit. Condom use is more frequent in the younger women age group of 15–24 years old, whereas the other variables are higher in different age groups.

South African women are more affected by HIV/AIDS than men, according to SADHS 2016 [21], with young females (15–24 years old) HIV prevalence four times higher than HIV prevalence among young males. Younger South African women carry the excessive burden of new HIV incidents due to sexual relationships with older males [28,29]. Among intergenerational sex, when young individuals with less sexual exposure are sexually connected with adults whose has higher rates of HIV infection, there is a high probability of significant power disparity, and condoms are less likely to be used [29]. Two studies [8,30] confirm that young women with less autonomy are significantly less likely to use condoms frequently. A study in Botswana, South Africa [31] reveals that women who are abused by their partner, financially dependent on their partner, or in a relationship with much older men are less likely to suggest condom use to their partner partners. In Uganda and Zimbabwe [32], women/wives also struggle to negotiate the use of condoms with their husbands/partners, which is due to financial dependency on the husband, or due to gender inequality leading to excluding women from the household decision-making process and consequently excluding them from decision-making involvement in sexual issues.

A study in three states in India [19] agrees with the findings of this study. Indian women with greater decision-making power are more likely to have more HIV and condom knowledge and to use condoms. Another study [16] reported that Nepalese married women with high autonomy are more likely to refuse sex or ask for condom use in risky situations- when their husband has STI or had an extramarital relationship- which may prevent new HIV/AIDS transmission.

A study in Cambodia [33] contradicts the findings of this study as they established that married Cambodian women with high decision-making power regarding their healthcare decisions and family visits decisions were (14%, and 17%, respectively) less likely to ask their partner to use condoms. The authors added that participation in decision-making for women led them to trust their partners and compensate for their involvement in risky sexual behavior. Other studies also show that women with high autonomy have more ability to make decisions regarding their reproductive health [14,17,18]. This study also agrees with another study [32] finding that reading newspapers is significantly associated with condom use.

A study among South African couples [34] claims that couples avoid discussing HIV prevention measures and risk evaluation due to their fear that the conversation will shift to be about trust and accusations of infidelity, which can escalate to violence. That fear impacts the couple’s ability to openly discuss condoms use or HIV testing discuss condom use or HIV testing. For couples who get tested, Knowledge of HIV status can be empowering. However, women with law autonomy may find it challenging to act upon their HIV status knowledge due to social norms and traditions [35]. In some cases, the situation might escalate to sexual intimate partner violence. However, a study in Ghana [36] showed that married women who got tested for HIV are more likely to be able to ask their husbands to use condoms; those women also tend to be wealthier and more educated; both factors positively affect women’s empowerment.

This study found a statistically significant association between women’s decision-making power and the use of HIV test kits, and contrasting results were observed among pregnant women in Kenya and their partners [37]. The difference between the findings can be since study [37] couples who were all expecting a baby, and their use for the HIV test kit might be due to their desire to protect their child, which is not the case of the sample of our current study that only explores women’s behavior regardless of their pregnancy status. A recent review reported that HIV test kit users’ high acceptability of HIV test kits was due to their convenience and privacy, and the instructions of the kit were easy to read and perform. However, users were concerned about the results’ pre-and post-test counseling accuracy and the test cost [38]. A study conducted in KwaZulu-Natal, South Africa [39] agrees with Stevens et al. [38], and it also reported that women find HIV test kits empowering and can provide autonomy. Stevens et al. [38] also noted that higher education levels and high financial status are among the factors that increased the ability to perform the test. That can explain the finding of this study that the use of HIV test kits is linked to wealth index, exposure to media, and education status. Additionally, the HIV test kit might be more convenient for working women than attending an appointment for regular testing at a clinic, and the kits also might be more available in urban areas than rural areas.

High education is a sign of women’s empowerment. Additionally, in this study, education level was positively associated with using all four preventive measures for HIV. A study in South Africa concurs with the findings as they established that after adjusting other variables, high education level is associated with sexual relationship power equity between partners [40].

This study found a statistically significant association between high decision-making power and with prevention of mother-to-child transmission (PMTCT) antiretroviral(ARV) drug use during pregnancy. Woman’s fertility desire and their HIV status is critical to meeting their needs for contraceptive methods. Research has shown that positive HIV status increases women’s fertility desire when they are on ART therapy or women who do not have children [26]. Another South African study revealed that ARV drug use was noticed among antenatal and postnatal women for PMTCT [41]; however, another study conducted in Zambia found no association between decision-making power and ARV drug use [42]

Additionally, women who made joint decisions with partners/others regarding their healthcare issues are five times more likely to use antiretroviral drugs during pregnancy than women who take decisions solely. Peltzer, Sikwane, and Majaja (2011) studied the factors that affect South African pregnant women’s adherence to antiretroviral drug therapy and found that the involvement of males in the treatment process is significantly associated with treatment adherence, in addition to low experience of discrimination and high HIV status disclosure [41]. The study finding also revealed that women who have support regarding healthcare issues and make their decisions jointly are likely to undertake HIV drugs to prevent transmission during pregnancy.

The findings of this study contradict that of a survey conducted in Zambia [42], which found no statistically significant associations between women’s decision-making power (own healthcare, child healthcare, household purchase, children school) and antiretroviral use during pregnancy as PMTCT, the authors explained that the lack of association could be because the therapy is being broadly and commonly used among the sample of their study.

## 5. Conclusions

In this study, we have explored decision-making power using five different domains (contraceptives, healthcare, large household purchase, visiting family or relatives, and spending earnings). Depending on those domains, the decision-making power “autonomy was calculated. Although all HIV preventive measures under study were statistically significantly associated with the high decision-making power, the use of condoms was the most important factor, and it was also found that women with high autonomy were three times more likely to use condoms. The literature supported this study’s finding, highlighting how women struggle to negotiate with their partners. This study also reflects on the importance of women’s empowerment as an approach to HIV prevention campaigns.

## Figures and Tables

**Table 1:** Socio-demographic determinants of HIV prevention behavior among the women of South Africa.

Variables	Frequency	Percentage	Condom Use	Tested for HIV	Drugs Used to Avoid Transmission	Use of HIV Test Kits
Age						
15–24	2913	34.2	1	1	1	1
25–34	2692	31.6	0.58 ( 0.51–0.66) ***	5.73 (4.84–6.84) ***	2.64 (2.02–3.46) ***	1.17 (1.01–1.34) *
35–44	1996	23.4	0.58 (0.50–0.67) ***	5.40 (4.42–6.59) ***	1.99 (1.50–2.63) ***	1.10 (0.94–1.30)
45+	913	10.7	0.46 (0.38–0.59) ***	3.24 (2.56–4.09) ***	1.53 (1.09–2.20) *	0.86 (0.69–1.07)
Mean ± SD	30.21 ± 9.86					
Place of residence						
Urban	4805	56.4	1	1	1	1
Rural	3709	43.6	0.85 (0.75–0.96) ***	0.84 (0.71–0.98) *	0.84 (0.65–1.07)	0.78 (0.68–0.89) ***
Educational Status						
No education	190	2.2	1	1	1	1
Primary	862	10.1	1.32 (0.87–1.99)	1.49 (0.99–2.25)	1.21 (0.63–2.31)	0.90 (0.52–1.52)
Secondary	6581	77.3	1.84 (1.25–2.72) ***	2.39 (1.61–3.54) ***	1.49 (0.81–2.75)	1.35 (0.82–2.22)
Higher	881	10.3	1.87 (1.22–2.85) ***	4.16 (2.59–6.70) ***	3.58 (1.64–7.78) ***	2.77 (1.65–4.65) ***
Ethnicity						
Black/African	7359	86.4	1	1	1	1
White	214	2.5	0.22 (0.14–0.35) ***	0.46 (0.31–0.69) ***	0.45 (0.25–0.81) **	0.80 (0.58–1.11)
Coloured	848	10.0	0.33 (0.27–0.40) ***	0.98 (0.78–1.23)	0.44 (0.25–0.81) ***	0.78 (0.65–0.94) **
Indian/Asian	88	1.0	0.29 (0.15–0.57) ***	0.41 (0.24–0.70) ***	2.12 (0.50–8.91)	0.87 (0.54–1.40)
Other	5	0.1	0.34 (0.04–3.08)	0.10 (0.02–0.65) *	0.04 (0.00–0.47) **	0.74 (0.06–9.30)
Wealth index						
Poorest	1763	20.7	1	1	1	1
Poorer	1865	21.9	1.03 (0.87–1.21)	1.17 (0.96–1.42)	0.96 (0.71–1.29)	1.41 (1.13–1.74) **
Middle	1956	23.0	1.13 (0.95–1.35)	1.21 (0.98–1.50)	1.21 (0.85–1.70)	1.69 (1.36–2.09) ***
Richer	1733	20.4	1.18 (0.97–1.45)	0.96 (0.75–1.22)	1.30 (0.88–1.92)	2.05 (1.63–2.59) ***
Richest	1197	14.1	0.83 (0.65–1.05)	0.60 (0.47–0.78) ***	0.83 (0.54–1.30)	2.40 (1.83–3.08) ***
Employment status						
No	5774	67.8	1	1	1	1
Yes	2740	32.2	1.12 (1.00–1.26) *	2.06 (1.71–2.47) ***	1.34 (1.04–1.72) *	1.50 (1.32–1.70) ***
Reading newspaper or magazine						
Not at all	3233	38.0	1	1	1	1
Less than once a week	2236	26.3	1.12 (1.02–1.34) *	1.09 (0.91–1.28)	1.25 (0.96–1.62)	1.40 (1.19–1.62) ***
At least once a week	3045	35.8	1.22 (1.10–1.40) **	1.05 (0.89–1.24)	1.30 (1.00–1.70) *	1.80 (1.54–2.08) ***
Listening to radio						
Not at all	2649	31.1	1	1	1	1
Less than once a week	1390	16.3	1.01 (0.85–1.20)	0.87 (0.71–1.05)	1.12 (0.82–1.54)	1.13 (0.92–1.34)
At least once a week	4475	52.6	1.06 (0.93–1.20)	1.07 (0.92–1.25)	1.10 (0.87–1.41)	1.36 (1.17–1.57) ***
Watching television						
Not at all	1549	18.2	1	1	1	1
Less than once a week	823	9.7	1.19 (0.95–1.48)	0.98 (0.76–1.26)	0.72 (0.49–1.07)	1.35 (1.05–1.74) *
At least once a week	6142	72.1	1.08 (0.92–1.27)	0.97 (0.80–1.17)	0.91 (0.67–1.23)	0.99 (0.81–1.22)

* statistically significant (*p* < 0.05); ** statistically significant (*p* < 0.01); *** statistically significant (*p* < 0.001).

**Table 2:** Relationship between decision-making power and HIV prevention behavior among women of South Africa.

Decision Making Score	Condom Use	Tested for HIV	Drugs Used to Avoid Transmission	Use of HIV Test Kits
Decision-making power				
Low	1	1	1	1
Moderate	0.84 (0.52–1.35)	1.24 (0.48–3.20)	0.58 (0.25–1.34)	0.70 (0.45–1.05)
High	3.05 (2.60–3.58) ***	0.24 (0.18–0.33) ***	0.56 (0.39–0.80) **	0.51 (0.44–60) ***

** statistically significant (*p* < 0.01); *** statistically significant (*p* < 0.001).

**Table 3:** Relationship between HIV prevention behavior and autonomy of women in South Africa.

Decision-Making Variables	Frequency (%)	Condom Use	Tested for HIV	Drugs Used to Avoid Transmission	Use of HIV Test Kits
**Decision makers for contraception**					
Respondent Alone	589 (38.5%)	1	1	1	1
Husband or partner someone else or others	179 (11.7%)	0.92 (0.52–1.62)	0.87 (0.26–2.86)	2.22 (0.44–11.28)	1.29 (0.73–2.28)
Respondent and Husband/partner	760 (49.8%)	0.99 (0.68–1.44)	1.33 (0.59–3.01)	1.24 (0.47–3.26)	1.38 (0.94–2.01)
**Person who decides on the respondent’s health care**					
Respondent Alone	1068 (37.7%)	1	1	1	1
Husband or partner someone else or others	186 (6.6%)	0.96 (0.39–2.38)	1.74 (0.19–18.06)	0.43 (0.09–1.98)	0.49 (0.18–1.39)
Respondent and Husband/partner	1576 (55.7%)	0.95 (0.62–1.45)	0.41 (0.15–1.06)	5.17 (1.46–18.24) **	1.09 (0.72–1.67)
**Person who usually decides on large household purchases**					
Respondent Alone	574 (20.3%)	1	1	1	1
Husband or partner someone else or others	221 (7.8%)	0.58 (0.23–1.47)	0.29 (0.07–1.18)	5.32 (0.42–67.42)	0.68 (0.26–1.81)
Respondent and Husband/partner	2035 (71.9%)	0.72 (0.45–1.14)	1.35 (0.47–3.91)	0.64 (0.21–1.97)	0.88 (0.55–1.41)
**Person who usually decides on visits to family or relatives**					
Respondent Alone	723 (25.5%)	1	1	1	1
Husband or partner someone else or others	181 (6.4%)	0.97 (0.40–2.40)	0.62 (0.11–3.58)	0.18 (0.03–0.88) *	1.84 (0.76–4.45)
Respondent and Husband/partner	1926 (68.1%)	0.94 (0.60–1.46)	0.69 (0.25–1.95)	0.83 (0.27–2.50)	1.04 (0.65–1.64)
**Person who usually decides how to spend respondents’ earnings**					
Respondent Alone	386 (31.4%)	1	1	1	1
Husband or partner someone else or others	69 (5.6%)	0.80 (0.39–1.86)	6.17 (0.67–57.06)	0.34 (0.05–2.24)	0.67 (0.28–1.62)
Respondent and Husband/partner	776 (63.0%)	0.99 (0.65–1.54)	2.41 (0.97–6.05) *	0.95 (0.33–2.75)	0.88 (0.57–1.36)

* Statistically significant (*p* < 0.05); ** statistically significant (*p* < 0.01).

## Data Availability

Available on request from the corresponding author.

## References

[1] Edwards L. (2007). HIV/AIDS, gender and sexuality: Socio-cultural impediments to women’s sexual and reproductive autonomy. Unravelling Taboos: Gender and Sexuality in Namibia.

[2] United Nation Population Fund UNFPA Programme of Action-Adopted. In Proceedings of the International Conference on Population and Development, Cairo, Egypt, 5–13 September 1994. https://www.unfpa.org/sites/default/files/event-pdf/PoA_en.pdf.

[3] United Nation Population Fund UNFPA (2019). Sexual and Reproductive Health and Rights: An Essential Element of Universal Health Coverage Background Document for the Nairobi Summit on ICPD25—Accelerating the Promise.

[4] Chapagain M. (2006). Conjugal power relations and couples’ participation in reproductive health decision-making: Exploring the links in Nepal. Gend. Technol. Dev..

[5] Price N.L., Hawkins K. (2007). A conceptual framework for the social analysis of reproductive health. J. Health Popul. Nutr..

[6] Granek L., Nakash O. (2017). The impact of militarism, patriarchy, and culture on Israeli Women’s reproductive health and well-being. Int. J. Behav. Med..

[7] Gilbert L., Walker L. (2002). Treading the path of least resistance: HIV/AIDS and social inequalities—A South African case study. Soc. Sci. Med..

[8] Pettifor A.E., Measham D.M., Rees H.V., Padian N.S. (2004). Sexual power and HIV risk, South Africa. Emerg. Infect. Dis..

[9] Wyatt G.E., Carmona J.V., Loeb T.B., Guthrie D., Chin D., Gordon G. (2000). Factors affecting HIV contraceptive decision-making among women. Sex Roles.

[10] Osuafor G.N., Maputle S.M., Ayiga N. (2018). Factors related to married or cohabiting women’s decision to use modern contraceptive methods in Mahikeng, South Africa. Afr. J. Prim. Health Care Fam. Med..

[11] Ogunjuyigbe P.O., Adeyemi E.O. (2005). Women’s sexual control within conjugal union: Implications for HIV/AIDS infection and control in a metropolitan city. Demogr. Res..

[12] Carroll J.J., Ngure K., Heffron R., Curran K., Mugo N.R., Baeten J.M. (2016). Gendered differences in the perceived risks and benefits of oral PrEP among HIV-serodiscordant couples in Kenya. AIDS Care.

[13] Dyson T., Moore M. (1983). On kinship structure, female autonomy, and demographic behavior in India. Popul. Dev. Rev..

[14] Acharya D.R., Bell J.S., Simkhada P., Van Teijlingen E.R., Regmi P.R. (2010). Women’s autonomy in household decision-making: A demographic study in Nepal. Reprod. Health.

[15] Orubuloye I.O., Oguntimehin F., Sadiq T. (1997). Women’s role in reproductive health decision making and vulnerability to STD and HIV/AIDS in Ekiti, Nigeria. Health Transit. Rev..

[16] Atteraya M.S., Kimm H., Song I.H. (2014). Women’s autonomy in negotiating safer sex to prevent HIV: Findings from the 2011 Nepal demographic and health survey. AIDS Educ. Prev..

[17] Sumri H.H. (2015). A National Study: The Effect of Egyptian Married Women’s Decision-Making Autonomy on the use of Modern Family Planning Methods. Afr. J. Reprod. Health.

[18] Do M., Kurimoto N. (2012). Women’s empowerment and choice of contraceptive methods in selected African countries. Int. Perspect. Sex. Reprod. Health.

[19] Bloom S.S., Griffiths P.L. (2007). Female autonomy as a contributing factor to women’s HIV-related knowledge and behaviour in three culturally contrasting states in India. J. Biosoc. Sci..

[20] Jewkes R.K., Dunkle K., Nduna M., Shai N. (2010). Intimate partner violence, relationship power inequity, and incidence of HIV infection in young women in South Africa: A cohort study. Lancet.

[21] National Department of Health (NDoH), Statistics South Africa (Stats SA), South African Medical Research Council (SAMRC), ICF (2019). South Africa Demographic and Health Survey 2016.

[22] UNAIDS (2020). South Africa. https://www.unaids.org/en/regionscountries/countries/southafrica.

[23] The South African National AIDS Council SANAC (2017). South Africa’s National Strategic Plan for HIV, TB and STIs 2017–2022.

[24] Holmes K.K., Levine R., Weaver M. (2004). Effectiveness of condoms in preventing sexually transmitted infections. Bull. World Health Organ..

[25] Senarath U., Gunawardena N.S. (2009). Women’s autonomy in decision making for health care in South Asia. Asia Pac. J. Public Health.

[26] Marlow H.M., Maman S., Groves A.K., Moodley D. (2012). Fertility intent and contraceptive decision-making among HIV positive and negative antenatal clinic attendees in Durban, South Africa. Health Care Women Int..

[27] Pinkerton S.D., Abramson P.R. (1995). Decision making and personality factors in sexual risk-taking for HIV/AIDS: A theoretical integration. Personal. Individ. Differ..

[28] UNAIDS (2017). A Snapshot of Men and HIV in South Africa.

[29] Muula A.S. (2008). HIV infection and AIDS among young women in South Africa. Croat. Med. J..

[30] Moyo W., Levandowski B.A., MacPhail C., Rees H., Pettifor A. (2008). Consistent condom use in South African youth’s most recent sexual relationships. AIDS Behav..

[31] Langen T.T. (2005). Gender power imbalance on women’s capacity to negotiate self-protection against HIV/AIDS in Botswana and South Africa. Afr. Health Sci..

[32] Mumtaz Z., Slaymaker E., Salway S. (2005). Condom use in Uganda and Zimbabwe: Exploring the influence of gendered access to resources and couple-level dynamics. A Focus on Gender: Collected Papers on Gender Using DHS Data.

[33] Ung M., Boateng G.O., Armah F.A., Amoyaw J.A., Luginaah I., Kuuire V. (2014). Negotiation for safer sex among married women in Cambodia: The role of women’s autonomy. J. Biosoc. Sci..

[34] Parker L., Pettifor A., Maman S., Sibeko J., MacPhail C. (2014). Concerns about partner infidelity are a barrier to adoption of HIV-prevention strategies among young South African couples. Cult. Health Sex..

[35] Tabana H., Doherty T., Rubenson B., Jackson D., Ekström A.M., Thorson A. (2013). ‘Testing together challenges the relationship’: Consequences of HIV testing as a couple in a high HIV prevalence setting in rural South Africa. PloS ONE.

[36] Tenkorang E.Y. (2012). Negotiating safer sex among married women in Ghana. Arch. Sex. Behav..

[37] Vrana-Diaz C.J., Korte J.E., Gebregziabher M., Richey L., Selassie A., Sweat M., Gichangi A. (2019). Relationship Gender Equality and Couples’ Uptake of Oral Human Immunodeficiency Virus Self-Testing Kits Delivered by Pregnant Women in Kenya. Sex. Transm. Dis..

[38] Stevens D.R., Vrana C.J., Dlin R.E., Korte J.E. (2018). A global review of HIV self-testing: Themes and implications. AIDS Behav..

[39] Harichund C., Moshabela M., Kunene P., Abdool Karim Q. (2019). Acceptability of HIV self-testing among men and women in KwaZulu-Natal, South Africa. AIDS Care.

[40] Closson K., Dietrich J.J., Beksinska M., Gibbs A., Hornschuh S., Smith T., Smit J., Gray G., Ndung’u T., Brockman M. (2019). Measuring sexual relationship power equity among young women and young men South Africa: Implications for gender-transformative programming. PLoS ONE.

[41] Peltzer K., Sikwane E., Majaja M. (2011). Factors associated with short-course antiretroviral prophylaxis (dual therapy) adherence for PMTCT in Nkangala district, South Africa. Acta Paediatr..

[42] Ford C., Chibwesha C.J., Winston J., Jacobs C., Lubeya M.K., Musonda P., Stringer J.S., Chi B.H. (2018). Women’s decision-making and uptake of services to prevent mother-to-child HIV transmission in Zambia. AIDS Care.

